# Falls and Associated Factors among Adolescents and Young Adults with Arthrogryposis Multiplex Congenita

**DOI:** 10.23937/2643-4571/1710035

**Published:** 2021-09-06

**Authors:** Jaclyn Megan Sions, Maureen Donohoe, Emma Haldane Beisheim, Tracy Michele Shank, Louise Reid Nichols

**Affiliations:** 1 Department of Physical Therapy, University of Delaware, USA; 2 Department of Therapeutic and Rehabilitative Services, Nemours Alfred I. DuPont Hospital for Children, USA; 3 Department of Orthopedics, Nemours Alfred I. DuPont Hospital for Children, USA

**Keywords:** Accidental falls, Arthrogryposis, Orthopedics, Patient reported outcomes measures, Pediatrics

## Abstract

**Background:**

Falls research among individuals with arthrogryposis multiplex congenita (AMC), a group of congenital conditions characterized by joint contractures in two or more body regions, is sparse. The primary objectives of this study were to estimate the prevalence of single, multiple, and injurious falls among adolescents and adults with AMC and identify factors associated with multiple and injurious falls.

**Methods:**

Individuals, aged 10–50 years, with a diagnosis of AMC completed questionnaires obtaining demographic and AMC-specific information, falls history (e.g., number, injurious/non-injurious), markers of bone health, orthopedic surgical history, and mobility aid use, as well as the Gillette Functional Assessment Questionnaire and the Saltin-Grimby Physical Activity Level Scale. Falls were defined as “any body part above the ankle coming to rest on the ground, floor, or a lower level”. Falling was defined as ≥ 1 fall, while multiple falls were defined as ≥ 2 falls in the past year.

Differences in falling, multiple falls, and injurious falls rates between adolescents (10–17 years) and adults (aged 18–50 years) were evaluated. Using univariate binary logistic regression models, associations between participant characteristics and multiple falls, as well as injurious falls, were evaluated, while considering age as a covariate (p ≤ 0.050); odds ratios (ORs) and 95% confidence intervals (CIs) were calculated.

**Results:**

Adolescents (N = 28; median age = 14 years) and adults (N = 40; median age = 32 years) with AMC had similar falling, i.e., 89.3% versus 70.0%, and injurious fall rates, i.e., 32.1% versus 27.5%, respectively (p > 0.050).

Adolescents with AMC, however, were more likely to report multiple falls in the past year, i.e., 89.3%, when compared to adults with AMC, i.e., 57.5% (p = 0.005). Beyond age, multiple falls were associated with a greater number of lower-limb surgeries [p = 0.036, OR (95%CI): 1.18 (1.01–1.39)], ability to transfer floor-to-stand with support [p = 0.002, OR (95%CI): 8.98 (2.30–35.06)], and increased mobility per the Gillette Functional Assessment Questionnaire [p = 0.004, OR (95%CI): 1.48 (1.13–1.92)]. Factors associated with a reduced odds of multiple falls were spinal involvement [p = 0.025, OR (95%CI): 0.23 (0.07–0.84)], history of spinal surgery [p = 0.018, OR (95%CI: 0.18 (0.04–0.74)], greater upper extremity involvement [OR (95%CI): 0.65 (0.44–0.95)], home assistive device use [p = 0.010, OR (95%CI): 0.15 (0.03–0.63)], and community wheelchair use [p = 0.006, OR (95%CI): 0.16 (0.04–0.59)]. None of the explored characteristics were associated with injurious falls in the past year (p > 0.050).

**Conclusion:**

Falls are exceedingly common among adolescents and adults with AMC; potential risk and protective factors for multiple falls are identified for future prospective falls research.

## Introduction

Arthrogryposis Multiplex Congenita (AMC) is a group of congenital conditions characterized by joint contractures in two or more body regions [1], occurring in approximately 1 in 5,000 to 10,000 live births, without a sex-predilection [2]. The majority of AMC cases are idiopathic, but mutations in over 400 different genes have also been associated with AMC [3]. Clinically, individuals with AMC present with joint stiffness, reduced muscle mass with increased fibrotic tissue and intramuscular fat, and normal to above-normal intellectual capabilities (unless the central nervous system is involved) [2,4]. Given the heterogeneity of joint involvement with AMC [5], functional consequences, including energy expenditure requirements for activities-of-daily living, mobility limitations, and participation restrictions, are quite variable [1,6].

Lower extremity alignment is compromised among individuals with AMC. With respect to joint involvement, 80–90% of individuals have clubfoot [7], while 30–90% [8] and 55–90% [9] have knee and hip involvement, respectively. Treatment of lower-limb deformities typically occurs distally to proximally, requiring multiple surgeries, particularly during early childhood [9]. Persistent knee contractures are particularly problematic and have been associated with non-ambulatory status [i.e., odds ratio (OR): 4.53] [10], given increased muscle activity requirements of the soleus, quadriceps, and hip extensors necessary to overcome increased dorsiflexion, knee and hip flexion moments resulting from the knee flexion contracture. As lower-extremity misalignment has been shown to increase fall risk [11], individuals with AMC, and particularly those with greater lower-extremity joint involvement, may be more apt to fall.

Falls are the leading cause of pediatric injury, accounting for the majority of emergency department injury visits [12,13]. As such, falls prevention is a critical area for injury prevention among adolescents, and particularly among adolescents with disabilities resulting in mobility limitations [14,15]. Among middle-aged adults in the general population, poor mobility and presence of musculoskeletal conditions are risk factors for falling and injurious falls [16,17]. Among adults with cerebral palsy, mobility decline occurs earlier in life (i.e., in young-to-middle adulthood) with concurrent deterioration in balance performance and elevation of fall risk [18]. Given young-to-middle aged adults with AMC are likely similarly at risk for early mobility-decline, it seems prudent to study falls among young-to-middle aged adults with AMC. Thus, the primary objectives of this exploratory study were to (a) Estimate the prevalence of single, multiple, and injurious falls among adolescents and young-to-middle aged adults with AMC and (b) Identify factors (i.e., characteristics) associated with multiple and injurious falls in the past year. We hypothesized single, multiple, and injurious fall rates among individuals with AMC, aged 10–50 years, would exceed rates reported in the general population, and that novel risk factors for multiple and injurious falls would be identified for consideration in future falls research in this patient population.

## Methods

### Participants

From March to July of 2019, adolescents and young-to-middle-aged adults with a medical diagnosis of AMC were recruited for this cross-sectional research study. Recruitment methods included print and online advertisements, as well as verbal recruitment. Individuals, aged 10–50 years, were considered for inclusion if they were English-speaking and -reading (due to lack of feasibility of having research staff fluent in other languages). Individuals over 50 years of age were excluded, as 51 years has been shown to be a critical age at which balance recovery and falls-avoidance is significantly decreased [19]. This study received approval from the Institutional Review Board for Human Subjects Research at the University of Delaware, and was conducted in accordance with the Declaration of Helsinki from the World Medical Association.

### Data collections

Following screening for eligibility and the informed consent/assent process, individuals completed a questionnaire regarding sex, race, ethnicity, age, markers of bone health (e.g., osteopenia, osteoporosis, history of lower-limb fracture, Vitamin D prescription), orthopedic surgical history, and lower-limb orthotic use. As assistive devise use is a clinically-useful indicator of future fall risk among older, community-dwelling adults [20,21], we also asked participants to report on home and community mobility aid use.

Individuals reported whether AMC occurred secondary to a known genetic cause, as well as the extent of body region involvement due to AMC. Individuals also reported the number of falls experienced in the past year, where a fall was defined as “any body part above the ankle coming to rest on the ground, floor, or a lower level [22]”. For the past year, individuals reported if they had (a) Any injurious falls, (b) Any falls requiring medical treatment, and (c) Any falls requiring hospitalization. Minors completed the paperwork with parental assistance.

Height and weight were obtained for calculation of body mass index (BMI). For adults, individuals were classified based on BMI: ‘underweight’ = < 18.5 kg/m^2^, ‘healthy’ = 18.5 to < 25 kg/m^2^, ‘overweight’ = 25 to < 30 kg/m^2^ and ‘obese’ = ≥ 30 kg/m^2^. Adolescents were classified using BMI percentiles (i.e., compared to sex- and age-matched peers): ‘underweight’ = < 5^th^ percentile; ‘healthy’ = 5^th^ to < 85^th^ percentile, ‘overweight’ = 85^th^ to < 95^th^ percentile and ‘obese’ = ≥ 95^th^ percentile, as per the Center for Disease Control and Prevention (https://www.cdc.gov/healthyweight/bmi/calculator.html).

To evaluate functional mobility, individuals were questioned regarding their ability to independently transfer floor-to-stand without and with external support (e.g., use of a chair), as might be necessary after a fall. Transferring floor-to-stand evaluates lower- extremity strength, flexibility, and postural control. Self-reported floor-to-stand transfer ability has been shown to be highly correlated to performance of floor-to-stand transfers [23]. Further, among older adults, inability to transfer floor-to-stand has been associated with an increased odds [OR (95% confidence interval, i.e., CI): 2.1 (1.1–3.9)] of serious falls-related injury [24]. The Gillette Functional Assessment Questionnaire, which has been shown to be reliable and valid among children with cerebral palsy and other central nervous system disorders [25], was used to evaluate typical walking ability while using an assistive device; 1 corresponds with ‘cannot take any steps at all’ and 10 corresponds with ‘walks, runs, climbs on level and uneven terrain without difficult or assistance’. The Saltin-Grimby Physical Activity Level Scale was used to evaluate leisure-time physical activity, where Level I = ‘physically inactive’ and Level IV = ‘regular hard physical training for competition sports’; concurrent and predictive validity have been previously established for this measure [26]. The Gillette Functional Assessment Questionnaire and/or Saltin-Grimby Physical Activity Level Scale were recompleted 1–10 days later in sample subsets to establish test-retest reliability for these measures among individuals with AMC.

### Statistical analyses

SPSS Statistics Version 26 (IBM Corp., Armonk, NY, USA) was used for all statistical analyses. Rates for falling, defined as ≥ 1 fall in the past year, and multiple falls, defined as ≥ 2 falls in the past year [27], were calculated for both adolescents (i.e., ages 10–17 years) and adults (i.e., ages 18–50 years). Age-group differences were evaluated using Chi Square Tests (or Fisher’s exact test) for nominal data (if the expected observations per category were < 5), Chi Square Tests for ordinal data, and independent t-tests, or Mann Whitney U Tests, for continuous data (as appropriate; p ≤ 0.050). Effect sizes were calculated when significant between-group differences were found (p ≤ 0.050). Test-retest reliability for the Gillette Functional Assessment Questionnaire and Saltin-Grimby Physical Activity Level Scale was evaluated using Cohen’s weighted kappa (k_w_).

Univariate binary logistic regression models were used to evaluate associations between participant characteristics and multiple falls (≥ 2 falls) in the past year (p ≤ 0.050). For nominal data, only independent variables with N ≥ 10 positive cases (~15% of sample) were included. Odds ratios (ORs) with 95% CIs were calculated for each independent variable.

## Results

Within the 5-month recruitment timeframe, we enrolled 68 individuals, i.e., 28 adolescents and 40 adults, with AMC. Table 1 and Table 2 provide sample characteristics. With respect to body anthropometrics (see Table 1), as expected, adults with AMC were taller (U = 661.5; *r* = 0.246), weighed more (U = 847.0; *r* = 0.552), and differed in BMI classification (p = 0.022), when compared to adolescents with AMC, with significantly more adolescents being classified as underweight per post-hoc testing (p = 0.027).

Bone health and orthopedic surgical history were similar between age groups (p > 0.050; Table 1). Overall, just over one-third of individuals had experienced a lower-limb fracture, and nearly 40% were prescribed vitamin D supplementation by a medical provider (Table 1). Lower-limb surgeries were more common than upper-limb and spinal surgeries (Table 1). The majority of participants, i.e., nearly 90%, reported AMC without a known genetic cause; just over 50% had spinal involvement, and the median number of involved limb regions was 12 out of 14 (Table 3), with the most commonly affected region being the foot/ankle. Table 3 provides detailed information regarding AMC-related regional limb involvement. When compared to adults, adolescents with AMC were more likely to wear bilateral lower-limb orthoses (p = 0.023; x^2^ = 5.180; w = 0.289).

There were no significant differences between adolescents and adults with AMC in history of falling, injurious falls, or falls requiring medical treatment (p > 0.050); no participants reported a fall requiring hospitalization (Table 1). Adolescents with AMC, however, were more likely to report multiple falls in the past year, i.e., 89.3%, when compared to adults with AMC, i.e., 57.5% (x^2^ = 8.015; p = 0.005), with a medium effect size (w = 0.343; Table 1).

**Figure 1:**
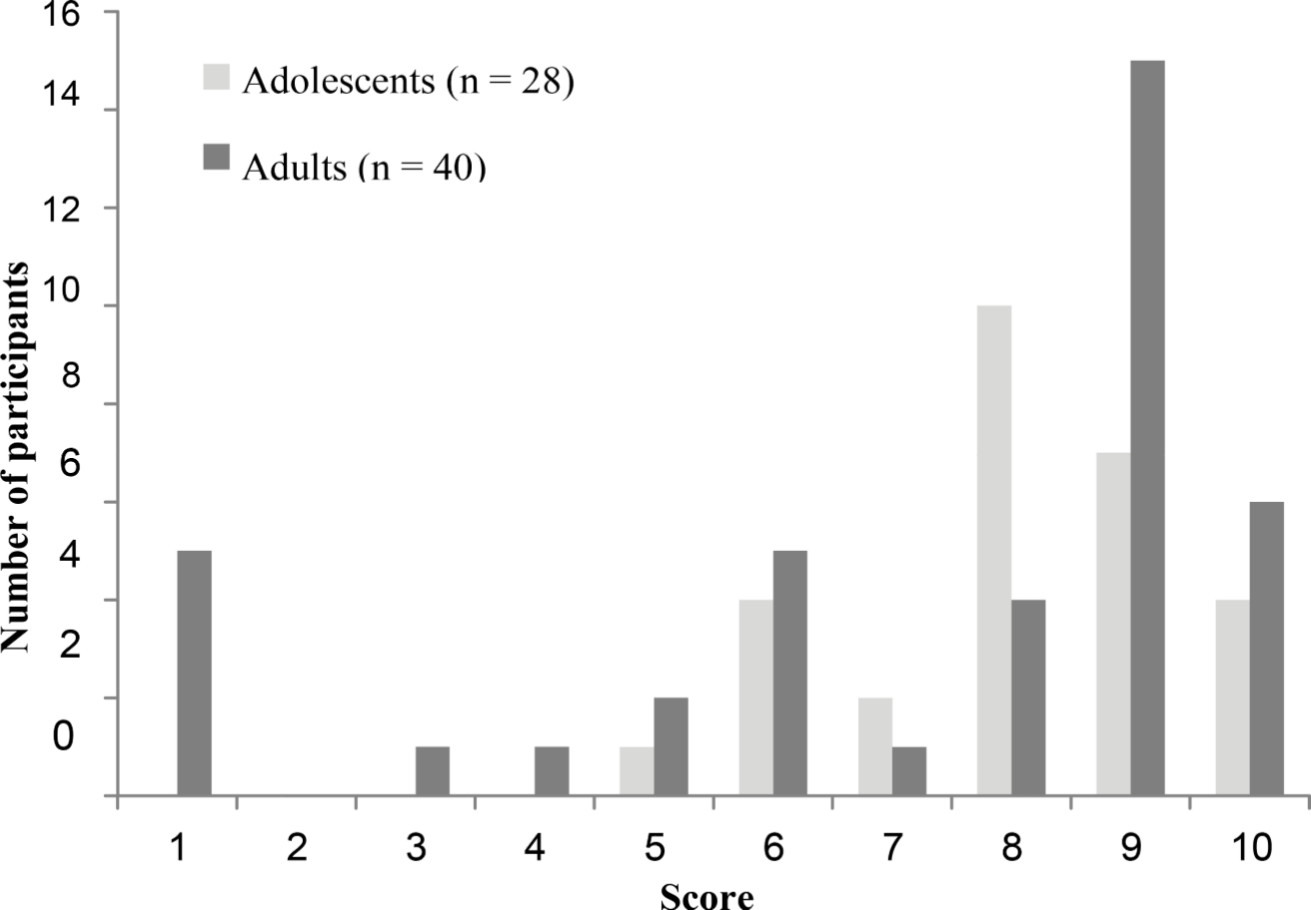
Gillette Functional Assessment Questionnaire Scores in Adolescents and Adults with AMC for the Total Sample (N = 68).

Self-reported floor-to-stand transfer ability was similar between age groups; only about 40% of participants could independently transfer without external support, but with external support (e.g., use of a chair), more than 75% were able (see Table 1). For test- retest reliability in the sample subsets, the Gillette Functional Assessment Questionnaire (n = 16) had substantial agreement (κw = 0.711; 95%CI: 0.470–.952; p < 0.001), as did the Saltin-Grimby Physical Activity Level Scale (n = 37; κw = 0.779; 95%CI: 0.638–0.921; p < 0.001). Table 2 contains reliability data for time-points 1 and 2 for the sample subsets. For the total sample, there were not significant differences between adolescents and adults with AMC for these two self-report measures (p = 0.735 for Gillette, Figure 1; p = 0.157 for Saltin-Grimby, Table 1). While 85.3% were ambulating at least short distances in the community (i.e., with a score of ≥ 6/10 on the Gillette Functional Assessment Questionnaire), only 14.7% reported the ability to walk, run, and climb on level and uneven terrain without difficulty or assistance (i.e., a score of 10/10; see Figure 1). Of the sample, 72.7% were not participating in regular physical activity of at least moderate intensity (see Table 1).

BMI classification was not significantly associated with multiple falls or injurious falls (p > 0.050), and was therefore, not considered as a covariate during univariate regression modeling. Univariate regression modeling (N = 67), indicated age, however, was a significant covariate for multiple falls [χ^2^ = 6.980, p = 0.011, OR (95%CI): 0.93 (0.88–0.98)], but not injurious falls [χ^2^ = 0.034, p = 0.854, OR (95%CI): 1.00 (0.95–1.05)].

As no participant characteristics were associated with injurious falls (p > 0.050), Table 4 is limited to regression model results for multiple falls, after accounting for age. In addition to younger age, the following three characteristics emerged as factors associated with higher odds of multiple falls: A greater number of lower-limb surgeries [OR (95%CI: 1.18 (1.01–1.39)], the ability to perform a floor-to-stand transfer with support [OR (95%CI): 8.98 (2.30–35.06)], and increased mobility per the Gillette Functional Assessment Questionnaire [OR (95%CI): 1.48 (1.13–1.92)]. Reduced odds of multiple falls was associated with spinal involvement [OR (95%CI): 0.23 (0.07–0.84)], spinal surgery [OR (95%CI): 0.18 (0.04–0.74)], greater upper-limb involvement [OR (95%CI): 0.65 (0.44–0.95)], home assistive device use [OR (95%CI): 0.15 (0.03–0.63)], and community wheelchair use [OR (95%CI): 0.16 (0.04–0.59)].

## Discussions

To our knowledge, this investigation is the first to estimate falls prevalence and to identify unique factors associated with multiple falls among individuals with AMC. Of the sample, 89% of adolescents and 70% of young adults with AMC reported falling and 32% and 28%, respectively, reported at least one injurious fall. While falling and injurious fall rates were similar among adolescents and young adults with AMC, the prevalence of multiple falls (≥ 2 falls in past year) was higher among adolescents.

Specifically, 89% of adolescents and 58% of young adults with AMC reported multiple falls. Data suggest approximately 50% of falls-related injuries were managed at home, while about 50% were managed in outpatient or emergency settings, as no participants reported a falls-related hospitalization. Collectively, our data suggest falls are a significant issue for both adolescents and young adults with AMC, warranting future falls-related investigations in this patient population.

Our findings emphasize the importance of not assuming falls-related risk factors identified in other populations will be risk factors among younger individuals with AMC. For example, prior work among older adults has identified female sex as a risk factor for falls and injurious falls [28,29], but female sex was not associated with multiple falls or injurious falls in our sample of adolescents and young adults with AMC. Increased age has been associated with a greater risk of falling and multiple falls among adults aged 18 years and older [21,29], but in our sample with AMC, younger (not older) age emerged as a factor associated with multiple falls in the past year. Furthermore, prior falls research among middle-aged adults suggests mobility limitations are associated with falls [16]. Conversely, we found better mobility among adolescents and young adults with AMC was associated with increased odds of reporting multiple falls.

Specifically, the ability to transfer floor-to-stand (with external support) and greater self-reported mobility per the Gillette Functional Assessment Questionnaire were associated with increased odds of reporting multiple falls. Further, we found reduced odds of multiple falls with home assistive device use and community wheelchair use (aids used to compensate for reduced mobility) among individuals with AMC. Inclusion of additional outcome measures (including performance-based measures) in future, prospective falls investigations may confirm our preliminary findings suggesting better mobility may be a risk factor for multiple falls among adolescents and young adults with AMC.

Why might better mobility be associated with increased odds of reporting multiple falls? It may be that individuals with greater functional capacities have greater exposure to higher-risk activities (e.g., stairs, running, vigorous activity) [30]. Future falls investigations in this patient population may consider incorporating body-worn accelerometers to obtain objective stair and step count data, allowing evaluation of relationships between high-risk activity exposures and falls. The use of accelerometers in future falls investigations among adolescents and adults with AMC may be further supported by a lack of associations between falls and self-reported physical activity level. In our study, physical activity level was classified with the Saltin-Grimby Physical Activity Level Scale, which we found to have acceptable test-retest reliability in this patient population, although activity captured with this self-report measure was not associated with multiple or injurious falls. Among younger, community-dwelling adults, Heijnen and colleagues similarly found physical activity levels (per the Leisure-Time Exercise Questionnaire) did not differentiate fallers from non-fallers, but suggested falls may be more prevalent as the frequency of activity increases [31]. Thus, accelerometers, which can objectively capture the frequency of activities, may be a more discriminant means of evaluating fall risk than self-reported physical activity.

With AMC, spinal deformities (e.g., curvatures), which may be present at birth or develop during childhood, are reported in up to 70% of individuals with AMC [1,32]. Spinal deformities may spontaneously fuse over time [33], limiting spinal mobility. As curvature progressions are often resistant to conservative management [34], it is not surprising that of our individuals with spinal involvement (N = 36), 36% (N = 13) reported undergoing spinal surgery. For individuals with AMC undergoing arthrodesis with instrumentation for curvature reduction (which has been reported in 25–40% of this population) [35], reduced spinal motion post-surgery may be expected. With reduced spinal mobility, either from spinal involvement alone or spinal involvement plus surgery, there may be reduced functional mobility, and consequently reduced fall risk. Future falls investigations may consider objectively assessing spinal range-of-motion among individuals with AMC to determine if spinal mobility is associated with functional mobility and/or fall risk.

Research among individuals with upper-limb loss (amputation) indicates heightened fall risk as compared to peers with intact limbs [36]. Heightened fall risk may be explained by a reduced ability to utilize upper-limb movements to aid in recovery from external perturbations, including slips and trips that may lead to falls [36]. Thus, with AMC, one would expect greater upper-limb involvement to be associated with multiple falls. In our sample, however, greater upper-limb involvement was associated with reduced odds of multiple falls. Such findings might be explained by participation limitations, as individuals with greater upper-limb involvement may have greater disability [35] resulting in reduced exposure to higher-risk activities [30]. It is also possible that individuals with AMC who have limited upper-limb movement from birth (due to extensive upper-limb involvement) compensate by reducing their whole body angular momentum range as a strategy to maintain postural stability during external perturbations; such perturbations require quick control of the body’s angular momentum to prevent a fall [37]. Evaluation of slips and trips in controlled environments (e.g., motion analysis laboratories), may enhance our understanding of how upper-limb involvement impacts postural stability (and may be related to falls) in this patient population.

Lack of significant differences in surgical data between adolescents and adults with AMC suggests most orthopedic surgeries are occurring in childhood, which is consistent with the premise that early, aggressive surgical treatment of AMC (to improve alignment and enhance mobility) improves short-term outcomes [9,38]. Among adults with AMC, however, studies evaluating the long-term impact of multiple pediatric orthopedic surgeries on functional mobility and falls are scarce. In our sample, the median number of lower-limb surgeries was 5 for both adolescents and young adults with AMC, which exceeds the number of total surgeries (i.e., 3, mostly lower-limb) reported among pediatric patients with cerebral palsy [39]. Furthermore, we found a greater number of lower-limb orthopedic surgeries to be associated with multiple falls, underscoring the importance of evaluating long-term outcomes (including fall-risk) following aggressive, lower-limb surgical management. While surgeries to improve lower-limb alignment have been shown to concurrently reduce trips and falls among adolescents (aged 12.5 ± 2.7 years) with Charcot-Marie-Tooth disease [40], surgical side-effects, such as lower-limb muscle atrophy and strength losses cannot be dismissed, since fall rates have been shown to decline with increasing muscle strength among adults [41]. Comprehensive muscle evaluations pre- and post-surgery among individuals with AMC may enable all stakeholders (e.g., patients, orthopedists, payers) to better assess the risk-to-benefit ratio for each surgery and the cumulative impact of multiple procedures. Yet, we would be remiss not to mention that the number of lower-limb surgeries might also be an indicator of AMC severity beyond that obtained from presence/absence of lower-limb joint involvement (which was not a factor associated with falls; see (Table 4). In this case, the number of lower-limb surgeries may be a consequence of severe lower-extremity misalignment, rather than a factor to mitigate to reduce fall risk.

Prevalence rates in this study for falling and multiple falls surpass rates reported in the general population and among other patient populations. For example, a large cohort study (N = 292), reported falls occurred in 18.5% of community-dwelling, younger adults (aged 20–45 years), [29] which is nearly 4 times less than the rate found among our younger adults, i.e., 70%. The prevalence of falls (70%) and multiple falls (58%) in our adults with AMC (median age: 32 years (25^th^, 75^th^ percentile: 24, 37) exceeds that reported in adults with upper-limb loss (mean age: 43 ± 17 years), i.e., 46% and 29%, respectively [36]. Among our younger adults with AMC, falling prevalence also exceeds the rate, i.e., 53%, reported among adults (mean age: 37 years) with cerebral palsy [42]. Our injurious falls rate, i.e., 29%, was similar to that reported among pediatric patients during inpatient hospital stays (32%), [43] and older, community-dwelling adults with severe knee osteoarthritis (29%) [44]. Importantly, while research on falls-related interventions exist for hospitalized pediatric patients [45] and community-dwelling, older adults [46], outpatient falls-prevention interventions are lacking for pediatric and young adult patients with mobility-limiting disabilities, such as AMC.

### Study Limitations

This study among adolescents and young adults with AMC expands on existing AMC research, which to-date has been largely pediatric-focused. But, given the cross-sectional nature of the study, we are unable to confirm that characteristics associated with multiple falls among adolescents and young adults with AMC are, in fact, risk (or protective) factors for future falls, which would require a prospective, cohort study design. In future longitudinal research, use of wearable sensors that accurately detect falls [47], may overcome known limitations with falls self- reporting, [22,48] particularly among adolescents, where falls are often attributed to ‘normal’ development and under-reported [49]. Further, reliance on participant self-report, without access to medical records, limited our ability to confirm data and/or capture more granular details, e.g., the genetic cause of AMC, falls-related injury type, specifics regarding surgical procedures, and prescribed medications [21].

In conclusion, this study indicates falling and multiple falls are significant and prevalent issues among adolescents and young adults with AMC, as rates exceed those of other patient populations. Younger age, a greater number of lower-limb orthopedic surgeries, and better functional mobility were associated with multiple falls among adolescents and young adults with AMC. Greater upper extremity involvement was associated with lower odds of reporting multiple falls in the past year, as was spinal involvement, a history of spinal surgery, home assistive device use, and community wheelchair use.

Unfortunately, no factors were identified that were associated with injurious falls, suggesting other variables (e.g., environmental, behavioral, medications) [29,50,51] may be important factors for subsequent falls-related inquiries. Future investigations may build on these cross-sectional findings by prospectively evaluating identified factors as predictors of (or protectors from) multiple falls, over time in a cohort of adolescents and adults with AMC. Consideration of the use of wearable accelerometers for falls detection and activity monitoring, in conjunction with self-report and performance-based mobility measures, is encouraged. Knowledge gained may ultimately enable development of effective falls-prevention interventions for individuals with AMC.

## Figures and Tables

**Table 1:** Participant characteristics.

	Total Sample	Adolescents	Adults	p

**Demographics**	N = 68	N = 28	N = 40	
Sex, Female*	47 (69.1)	17 (60.7)	30 (75.0)	0.210
Ethnicity, Non-Hispanic/Latino*	62 (91.2)	27 (96.4)	35 (87.5)	0.389
Race	N = 67	N = 28	N = 39	
White/Caucasian*	58 (86.6)	23 (82.1)	35 (89.7)	0.474

**Age**	N = 67	N = 28	N = 39	
Years^†^	21 (14, 33)	14 (12, 15)	32 (24, 37)	**< 0.001**

**Body Anthropometries**	N = 65	N = 27	N = 38	
Height, m^†^	1.52 (1.45, 1.61)	1.50 (1.42, 1.57)	1.57 (1.49, 1.63)	**0.048**
Weight, kg^†^	50.5 (40.8, 61.2)	41.3 (34.0, 49.0)	57.6 (51.2, 66.7)	**< 0.001**
Body Mass Index Classification*				**0.022**
Underweight	9 (13.8)	7 (25.0)	2 (5.3)	
Healthy	38 (58.5)	17 (63.0)	21 (55.3)	
Overweight	12 (18.5)	2 (7.4)	10 (26.3)	
Obese	6 (9.2)	1 (3.7)	5 (13.2)	

**Bone Health**	N = 68	N = 28	N = 40	
Osteopenia*	11 (16.2)	4 (14.3)	7 (17.5)	1.000
Osteoporosis*	4 (5.9)	1 (3.6)	3 (7.5)	0.638
History of Lower-limb Fracture*	23 (33.8)	8 (28.6)	15 (37.5)	0.444
Vitamin D Prescription*	27 (39.7)	12 (42.9)	15 (37.5)	0.657

**Orthopedic Surgical History**	N = 67	N = 28	N = 39	
Number of Upper-limb Surgeries^†^	1 (0, 3)	2 (0, 3)	0 (0, 2)	0.197
Number of Lower-limb Surgeries^†^	5 (3, 8)	5 (3, 7)	5 (2, 8)	0.660
Spinal Surgery*	13 (19.4)	5 (17.9)	8 (20.5)	0.786

**Mobility Aids**	N = 68	N = 28	N = 40	
Wheelchair use*				
Home Mobility	8 (11.8)	1 (3.6)	7 (17.5)	0.128
Community Mobility	19 (27.9)	7 (25.0)	12 (30.0)	0.651
No assistive device use*				
Home-Ambulation	56 (82.4)	24 (85.7)	32 (80.0)	0.748
Community-Ambulation	41 (60.3)	18 (64.3)	23 (57.5)	0.574

**Fall History**	N = 68	N = 28	N = 40	
≥ 1 fall*	53 (77.9)	25 (89.3)	28 (70.0)	0.078
≥ 2 falls*	48 (70.6)	25 (89.3)	23 (57.5)	**0.005**
Injurious fall*	20 (29.4)	9 (32.1)	11 (27.5)	0.679
Medical treatment necessary*	10 (14.7)	5 (17.9)	5 (12.5)	0.730
Hospitalization*	0 (0.0)	0 (0.0)	0 (0.0)	--

**Functional Mobility**	N = 68	N = 28	N = 40	
Floor-to-Stand Transfer without support*	27 (39.7)	14 (50.0)	13 (32.5)	0.147
Floor-to-Stand Transfer with support*	52 (76.5)	22 (78.6)	30 (75.0)	0.733

**Activity Level**				
Saltin-Grimby Physical Activity	N = 66	N = 26	N = 40	0.157
Level Scale*				
Level I	20 (30.3)	7 (26.9)	13 (32.5)	
Level II	28 (42.4)	8 (30.8)	20 (50.0)	
Level III	12 (18.2)	7 (26.9)	5 (12.5)	
Level IV	6 (9.1)	4 (15.4)	2 (5.0)	

* Data presented as N (% of sample)

† Data presented as median (25^th^, 75^th^ percentile).

**Table 2:** Prevalence Data for Test-Retest Reliability Analyses.

Level	Time-point 1	Time-point 2

**Gillette Functional Assessment Questionnaire (N = 16)** *		

6	2 (12.5)	2 (12.5)
7	0 (0.0)	0 (0.0)
8	3 (18.8)	4 (25.0)
9	6 (37.5)	6 (37.5)
10	5 (31.3)	4 (25.0)

**Saltin-Grimby Physical Activity Level Scale (N = 37)**		

I	14 (37.8)	14 (37.8)
II	13 (35.1)	14 (37.8)
III	7 (18.9)	7 (18.9)
IV	3 (8.1)	2 (5.4)

Data presented as N (% of sample).

* No participants reported 1–5 on the Gillette Functional Assessment Questionnaire.

**Table 3:** AMC-Specific characteristics.

	Total Sample (N = 68)	Adolescents (N = 28)	Adults (N = 40)	p
Genetic Cause*	8 (11.8)	6 (21.4)	2 (5.0)	0.057
Spinal Involvement*	36 (52.9)	13 (46.4)	23 (57.5)	0.368
Number of Upper-Limb Regions Affected (0–8)^†^	8 (4, 8)	8 (4, 8)	8 (5, 8)	0.718
Number of Lower-Limb Regions Affected (0–6)^†^	6 (3, 6)	6 (3, 6)	6 (3, 6)	0.995

* Data presented as N (% of sample).

† Data presented as median (25^th^, 75^th^ percentile).

**Table 4:** Characteristics associated with multiple falls after considering age as covariate.

	χ^2^	p	OR (95%CI)

**Female Sex** (N = 67)	0.802	0.369	1.74 (0.52–5.87)

**AMC-Specific Data**			
Spinal Involvement (N = 67)	5.630	**0.025**	0.23 (0.07–0.84)
Number of Upper-Limb Regions Affected (N = 67)	6.845	**0.027**	0.65 (0.44–0.95)
Number of Lower-Limb Regions Affected (n = 67)	1.601	0.224	0.81 (0.58–1.14)
Lower-Limb Orthotic Use (N = 63)	0.218	0.639	1.34 (0.40–4.50)

**Bone Health**			
Osteopenia (N = 67)	3.207	0.074	0.28 (0.07–1.13)
Vitamin D Prescription (N = 67)	0.015	0.903	1.08 (0.34–3.42)
History of Lower-Limb Fracture (N = 67)	3.822	0.051	0.31 (0.10–1.01)

**Orthopedic Surgical History**			
Number of Upper-Limb Surgeries (N = 66)	0.759	0.379	0.92 (0.76–1.11)
Number of Lower-Limb Surgeries (N = 66)	6.730	**0.036**	1.18 (1.01–1.39)
Spinal Surgery (N = 66)	5.907	**0.018**	0.18 (0.04–0.74)

**Mobility Aids**			
Wheelchair Use Community (N = 67)	8.348	**0.006**	0.16 (0.04–0.59)
Assistive Device Use Home (N = 67)	7.111	**0.010**	0.15 (0.03–0.63)
Community (N = 67)	3.097	0.082	0.36 (0.11–1.14)

**Functional Mobility**			
Floor-to-Stand Transfer without Support (N = 67)	1.125	0.297	1.92 (0.56–6.55)
Floor-to-Stand Transfer with Support (N = 67)	11.149	**0.002**	8.98 (2.30–35.06)
Gillette Functional Assessment Questionnaire (N = 67)	10.610	**0.004**	1.48 (1.13–1.92)

**Physical Activity**			
Saltin-Grimby Physical Activity Level Scale (N = 65)	0.385	0.539	1.23 (0.63–2.39)

Data is presented for the second block of each model after controlling for age in block 1. One adult participant was missing specific age data resulting in a maximum N = 67.

Abbreviations: χ^2^ = Chi Square test statistic for second block; OR = Odds Ratio; CI = Confidence Interval.

## Abbreviations

AMC: Arthrogryposis Multiplex Congenita
kw: Cohen’s Weighted Kappa
OR: Odds Ratio
CI: Confidence Interval
